# MicroRNA‐Mediated Regulation of Brain Aging Hallmarks: Implications for Neurodegeneration and Neural Recovery

**DOI:** 10.1002/brb3.71466

**Published:** 2026-05-05

**Authors:** Mustafa T. Ardah, Pareshkumar N. Patel, Omayma Salim Waleed, Jamuna K. V., Vandana Tripathi, L. Lakshmi, Rajashree Panigrahi, Manoj Kumar Mishra

**Affiliations:** ^1^ Faculty of Allied Medical Sciences, Hourani Center For Applied Scientific Research Al Ahliyya Amman University Amman Jordan; ^2^ Department of Pharmacy, Faculty of Pharmacy Gokul Global University Sidhpur Gujarat India; ^3^ Department of Anesthesia Techniques, Health and Medical Techniques College Alnoor University Mosul Iraq; ^4^ Department of Forensic Science, School of Sciences JAIN (Deemed to Be University) Bangalore Karnataka India; ^5^ Department of Pharmacy, Sharda School of Pharmacy Sharda University Greater Noida India; ^6^ Department of Nursing Sathyabama Institute of Science and Technology Chennai Tamil Nadu India; ^7^ Department of Microbiology, IMS and SUM Hospital Siksha O Anusandhan (Deemed to Be University) Bhubaneswar Odisha India; ^8^ Department of Natural Sciences Salale University Fitche Ethiopia

**Keywords:** brain aging, epigenetic regulation, microRNA, neurodegeneration, neural recovery

## Abstract

**Purpose:**

Aging is a progressive biological process characterized by the decline of cellular and tissue homeostasis, increasing vulnerability to chronic diseases, especially neurodegeneration. This review examines the role of microRNAs (miRNAs) in brain aging by linking them to the updated hallmarks of aging and key neurobiological processes.

**Method:**

This is an integrative literature review of studies on brain aging, neurodegeneration, and miRNA‐mediated regulation. Evidence was synthesized from transcriptomic analyses, experimental studies, and research on circulating systemic factors, including extracellular vesicle‐associated miRNAs and plasma proteins. The review focuses on miRNA involvement in genomic stability, nutrient sensing, proteostasis, autophagy, mitochondrial function, telomere maintenance, epigenetic regulation, and cellular senescence.

**Finding:**

miRNAs regulate multiple aging‐related pathways, including DNA repair, IGF‐1/AKT/mTOR signaling, proteostasis, autophagy, and mitochondrial homeostasis. In aging brains, transcriptomic data consistently show reduced expression of genes involved in synaptic plasticity, metabolism, and maintenance, alongside increased inflammatory and immune signaling. Circulating systemic factors and extracellular vesicle miRNAs also influence brain aging, affecting neuroplasticity, cognitive function, and regenerative capacity. These mechanisms contribute to neuroinflammation, synaptic dysfunction, myelin impairment, and neurovascular unit disruption.

**Conclusion:**

miRNAs are key regulators of brain aging and neurodegeneration and hold promise as biomarkers and therapeutic targets. However, clinical application is limited by disease heterogeneity, context‐dependent effects, off‐target activity, and challenges in central nervous system delivery. Further research is needed to support safe and effective miRNA‐based interventions.

## Graphical abstract

**Hallmarks of brain aging regulated by microRNAs (miRNAs)**. This graphical abstract illustrates the central role of miRNAs in coordinating key biological processes associated with brain aging. miRNAs regulate multiple interconnected hallmarks, including genomic instability, telomere attrition, epigenetic alterations, loss of proteostasis and impaired autophagy, dysregulated nutrient sensing, mitochondrial dysfunction, cellular senescence and stem cell exhaustion, neuroinflammation and altered intercellular communication, and neurovascular unit or blood–brain barrier dysfunction. These molecular and cellular changes collectively contribute to functional outcomes such as synaptic dysfunction, myelin disruption, increased neurodegenerative vulnerability, and impaired neural recovery or reduced plasticity. In addition, circulating factors and extracellular vesicle‐associated miRNAs may influence brain aging through systemic signaling mechanisms, further modulating neuronal resilience and regenerative capacity.

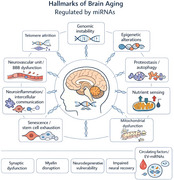

**Abbreviations**
AD: Alzheimer's disease
AKT: protein kinase B
APP: amyloid precursor protein
ATG: autophagy‐related gene/protein
Aβ: amyloid‐beta
BBB: blood–brain barrier
BER: base excision repair
CA1: cornu ammonis area 1
CCL11: C‐C motif chemokine ligand 11
CDS: coding sequence
CNS: central nervous system
DAMPs: damage‐associated molecular patterns
DNMT: DNA methyltransferase
DSBs: DNA double‐strand breaks
ER: endoplasmic reticulum
ETC: electron transport chain
Exp5 / Exportin‐5: exportin‐5
FEN1: flap endonuclease 1
FOXO: forkhead box O transcription factors
GDF11: growth differentiation factor 11
HDAC: histone deacetylase
HR: homologous recombination
HSP: heat shock protein
ICAM‐1: intercellular adhesion molecule 1
IFN‐γ: interferon gamma
IGF‐1: insulin‐like growth factor 1
IL‐1: interleukin‐1
LIG1: DNA ligase 1
lncRNA: long non‐coding RNA
LPS: lipopolysaccharide
MAPKs: mitogen‐activated protein kinases
miR/miRNA: microRNA
MREs: miRNA recognition elements
mRNA: messenger RNA
mTOR: mechanistic target of rapamycin
MYRF: myelin regulatory factor
NF‐κB: nuclear factor kappa B
NHEJ: non‐homologous end joining
NO: nitric oxide
NPAS4: neuronal PAS domain protein 4
PAMPs: pathogen‐associated molecular patterns
PARP1: poly(ADP‐ribose) polymerase 1
PNUTS / PPP1R10: protein phosphatase 1 regulatory subunit 10
Pol II: RNA polymerase II
pre‐miRNA: precursor microRNA
pri‐miRNA: primary microRNA transcript
PRRs: pattern recognition receptors
PTEN: phosphatase and tensin homolog
RAB5A: Ras‐related protein Rab‐5A
Ran‐GTP: Ran GTPase in the GTP‐bound form
RISC: RNA‐induced silencing complex
RNAi: RNA interference
ROS: reactive oxygen species
SASP: senescence‐associated secretory phenotype
SIRT1: sirtuin 1
SSBR: single‐strand break repair
TDP2: tyrosyl‐DNA phosphodiesterase 2
TERF1 / TERF2 (TRF1 / TRF2): telomeric repeat‐binding factor 1 / 2
TERT: telomerase reverse transcriptase
TLRs: toll‐like receptors
TNF‐α: tumor necrosis factor alpha
TOP2: topoisomerase II
tPA: tissue plasminogen activator
UDG: uracil‐DNA glycosylase
UTR (3′UTR / 5′UTR): untranslated region
UVRAG: UV radiation resistance‐associated gene
VCAM‐1: vascular cell adhesion molecule 1
VPS34: vacuolar protein sorting 34
XRCC1: X‐ray repair cross‐complementing protein 1

## 1 Introduction

Aging is a progressive biological process characterized by interconnected declines in cellular homeostasis, tissue resilience, and organ function, which together increase susceptibility to morbidity and mortality. These age‐associated changes contribute to a broad spectrum of chronic disorders, including cancer, metabolic disease, cardiovascular pathology, neurovascular dysfunction, and neurodegenerative conditions (Tenchov et al. 2024). Among the regulatory systems increasingly implicated in these processes, microRNAs (miRNAs) have emerged as an important post‐transcriptional layer capable of shaping multiple aging‐related pathways simultaneously. In this review, we adopt a brain‐centered perspective to examine major hallmarks of aging and to discuss representative miRNAs that have been linked to each hallmark and to aging biology more broadly (Sarkar et al. 2019).

From a mechanistic standpoint, aging is commonly organized into several interacting biological domains, including genomic instability, dysregulated nutrient sensing, loss of proteostasis, impaired autophagy, mitochondrial dysfunction, stem cell exhaustion, telomere attrition, epigenetic alteration, cellular senescence, and disrupted intercellular communication (Guha et al. 2018). These processes are highly interconnected rather than biologically isolated, and their convergence is particularly relevant in the aging central nervous system, where neuronal longevity, limited regenerative capacity, glial reactivity, and neurovascular integrity all influence vulnerability to degeneration. miRNAs are especially relevant in this context because a single miRNA can modulate multiple transcripts within the same pathway or across converging pathways, thereby amplifying relatively modest regulatory shifts into broader phenotypic consequences (Salmaninejad et al. 2022).

One influential line of evidence supporting the systemic regulation of aging comes from heterochronic parabiosis experiments, in which young and aged animals share a common circulation. These studies suggest that aged tissues can retain a degree of plasticity and regenerative responsiveness when exposed to a younger systemic environment (Rebo et al. 2016). In several experimental settings, transfer of young plasma into older animals has been associated with improvements in age‐related traits, including cardiac hypertrophy, synaptic dysfunction, learning and memory decline, muscle wasting, and reduced cerebral perfusion (Mendelsohn and Larrick 2016). Conversely, exposure of young animals to aged plasma can impair synaptic plasticity and worsen performance in selected learning and memory paradigms, indicating that aging is shaped not only by cell‐intrinsic deterioration but also by circulating systemic signals (Xiong et al. 2017).

Among the circulating factors proposed to mediate these effects, GDF11 and CCL11 have received particular attention. GDF11, a member of the TGF‐β superfamily, has been reported in some studies to decline with age and to exert rejuvenation‐associated effects when restored, including attenuation of age‐related cardiac hypertrophy (Rochette et al. 2015). By contrast, CCL11 has been linked to older systemic milieus and to poorer cognitive performance, and elevated levels have been detected in the plasma and cerebrospinal fluid of older adults (Huber et al. 2018). At the same time, the evidence remains incomplete and, in some cases, contested. The upstream regulation of these factors, their dominant tissue sources, and the reasons for their age‐associated changes are not yet fully resolved. It is therefore unlikely that a single circulating mediator can account for the full spectrum of systemic aging phenotypes (Mertz 2016).

Beyond soluble proteins, other circulating components may also contribute to age‐related tissue remodeling, including extracellular vesicles, non‐coding RNAs, and exosome‐associated miRNAs. Because organ structure and function are ultimately governed by gene‐expression programs, age‐dependent changes in transcriptional and post‐transcriptional regulation are likely to be closely tied to both physiological aging and organ‐specific pathology (Safaei et al. 2025). In the brain, this issue is especially important because gene‐regulatory instability can affect neuronal maintenance, glial activation states, synaptic function, and inflammatory tone at the same time (Cornell et al. 2022).

This review therefore focuses on brain‐relevant aging hallmarks and evaluates how miRNAs may participate in these processes across different levels of evidence, including prediction‐based, associative, and functionally validated observations. We discuss candidate miRNAs linked to genomic maintenance, metabolic signaling, proteostasis, inflammatory regulation, and cellular senescence, while also considering their relevance to neurodegeneration and neural recovery‐related responses.

## 2 Brain Aging and Gene‐Expression Pattern Shifts

Large‐scale transcriptomic profiling, including bulk tissue analyses and single‐cell or single‐nucleus approaches, has provided a detailed view of aging‐associated gene‐expression changes across multiple human brain regions, including the prefrontal cortex (Fröhlich et al. 2024). Rather than referring vaguely to changes occurring “around the 40s,” it is more accurate to state that several human lifespan datasets suggest a midlife‐to‐late‐life transition, often spanning approximately the fifth to seventh decades, during which age‐associated transcriptional divergence becomes increasingly evident, although the exact inflection point varies by cohort, brain region, and analytic platform (Ianov et al. 2016).

Genes that decline with advancing age are frequently enriched in pathways related to synaptic plasticity, myelin maintenance, mitochondrial metabolism, intracellular trafficking, learning and memory support, stress defense, DNA repair, and transcriptional regulation (Popa‐Wagner et al. 2026). These patterns suggest weakening of neural maintenance and adaptive capacity rather than a simple generalized reduction in gene activity, which may increase susceptibility to cognitive decline and neurodegenerative vulnerability (Qureshi and Mehler 2018).

By contrast, genes that increase with age are commonly associated with immune signaling, cytokine‐related pathways, glial activation, and inflammatory responses, a pattern widely discussed in relation to inflammaging (Pojero et al. 2022). This does not imply that all inflammatory activity is uniformly detrimental, since immune signaling can be context dependent and in some settings reparative. However, persistent low‐grade inflammatory activation in the aging brain is widely viewed as a contributor to synaptic dysfunction, impaired tissue homeostasis, and increased risk of neurodegenerative pathology (Müller and Di Benedetto 2026).

miRNA profiling studies in the aging cortex and cerebellum, including analyses in humans and closely related species, further suggest that age‐associated miRNA shifts may affect gene networks linked to cognitive decline and neurodegenerative risk (Arleo et al. 2024). Even so, many of these relationships remain associative rather than definitively causal, and the strength of evidence varies considerably across individual miRNA‐target pairs. This distinction is important when interpreting whether a given miRNA acts as a biomarker of brain aging, a byproduct of cellular stress, or a direct mechanistic contributor (Petracci et al. 2025). A major driver of age‐related changes in gene output is epigenetic regulation, including altered DNA methylation, histone modification, chromatin accessibility, and shifts in non‐coding RNA expression. miRNAs represent one component of this broader regulatory architecture (Saul and Kosinsky 2021). Although large numbers of putative miRNAs have been cataloged across species, not all detected sequences have equivalent functional support, and the number of biologically active miRNAs is likely smaller than raw database counts may suggest (Behl et al. 2024). For this reason, it is important to distinguish between detected miRNA species, differentially expressed candidates, and miRNAs supported by direct functional validation.

Despite these limitations, miRNAs have emerged as important regulators of senescence, stress signaling, mitochondrial function, inflammatory responses, and cell‐cycle control (Neault et al. 2017). In the sections that follow, we examine candidate aging hallmarks through a brain‐focused lens and discuss specific miRNAs that have been implicated in these pathways, with attention to evidence strength, mechanistic plausibility, and relevance to age‐related brain disorders. We also consider how miRNA‐associated inflammatory and stress pathways intersect with acute neural injury, such as stroke, and chronic neurodegenerative conditions, where aging‐related vulnerability may shape both disease progression and recovery capacity (Sarkar et al. 2019; Lupo et al. 2019).

## 3 MicroRNAs: Biogenesis From the Nucleus to the Cytoplasm

miRNAs are small endogenous non‐coding RNAs, typically about 22 nucleotides in length, which regulate gene expression primarily through interactions with target messenger RNAs. By promoting mRNA degradation and/or repressing translation, they reduce protein output at the post‐transcriptional level (O'Brien et al. 2018). Many miRNA genes are organized in chromosomal clusters, and some belong to conserved sequence families, such as the miR‐34 family. This family‐level organization is functionally relevant because closely related miRNAs may share overlapping target spectra, although their regulatory effects are not always identical across tissues or contexts (Cao et al. 2016).

Most miRNA genes are transcribed by RNA polymerase II, and in some cases by RNA polymerase III, as long primary transcripts known as pri‐miRNAs. In the nucleus, pri‐miRNAs are processed by the microprocessor complex, which includes Drosha and DGCR8, into shorter precursor miRNAs (pre‐miRNAs) (Lee et al. 2004). These hairpin‐shaped pre‐miRNAs are then exported to the cytoplasm, predominantly through Exportin‐5. In the cytoplasm, Dicer cleaves pre‐miRNA to generate an approximately 22‐nucleotide miRNA duplex with characteristic 2‐nucleotide 3′ overhangs; this duplex may also contain internal mismatches or bulges (Teoh et al. 2018). This structure should be distinguished from long exogenous double‐stranded RNA, which is processed through different biological contexts and can trigger distinct cellular responses.

Following Dicer processing, one strand of the duplex is preferentially incorporated into an Argonaute‐containing RNA‐induced silencing complex (RISC) and serves as the guide strand. The opposite strand, often referred to as the passenger strand or miRNA*, is commonly degraded. However, passenger strands are not necessarily nonfunctional, since in some settings they can also be loaded into Argonaute complexes and regulate target transcripts (Kim et al. 2020). Once incorporated into RISC, the guide miRNA directs the complex to target RNAs through base pairing, most often within the 3′ untranslated region, although binding to coding regions or other transcript segments can also occur. Depending on the degree of complementarity, the cellular environment, and the composition of the silencing machinery, this interaction may suppress translation and/or accelerate mRNA destabilization and decay (Chipman and Pasquinelli 2019). Because a single miRNA can regulate numerous transcripts simultaneously, relatively modest changes in miRNA abundance may influence entire molecular programs rather than isolated genes. This systems‐level property is especially relevant in aging, where stress‐responsive, metabolic, inflammatory, and genome‐maintenance pathways are already under pressure and may therefore be particularly sensitive to post‐transcriptional regulatory shifts (Meseguer‐Donlo et al. 2023; Steinkraus et al. 2016).

## 4 Genome Maintenance Declines With Age: DNA Repair Under Pressure

Cells experience DNA damage throughout life from endogenous sources, including metabolic by‐products and replication‐associated stress, as well as exogenous insults such as radiation and toxic exposures (Huang and Zhou 2021). To preserve genomic integrity, they rely on coordinated DNA damage signaling, cell‐cycle checkpoints, and multiple repair systems that detect lesions, pause cell‐cycle progression when needed, and restore DNA structure whenever possible. Even so, damage can accumulate over time, particularly when repair efficiency declines with age (Ahmed et al. 2023).

Different classes of DNA lesions are resolved by distinct repair pathways. These include base excision repair and single‐strand break repair (BER/SSBR), homologous recombination, non‐homologous end joining, mismatch repair, and translesion synthesis (Chatterjee and Walker 2017). Telomere maintenance should not be presented as a generic DNA repair pathway equivalent to homologous recombination or non‐homologous end joining. Instead, telomere integrity is preserved mainly through telomerase activity, shelterin‐mediated end protection, and, in some settings, alternative lengthening of telomeres. This distinction is important because telomeric ends can resemble DNA double‐strand breaks, yet shelterin normally prevents inappropriate activation of canonical DNA repair responses at chromosome termini (Baiken et al. 2020; Rembiałkowska et al. 2025). Across aging tissues, expression of several repair‐associated genes appears to decline, raising the possibility that age‐related changes in transcriptional and post‐transcriptional regulation contribute to progressive genome instability (Stifel et al. 2022).

This issue is particularly relevant in the aging brain. Neurons are long‐lived, highly metabolically active cells that operate under substantial oxidative burden, making them especially vulnerable to oxidized DNA bases, abasic sites, and damaged DNA ends. In this setting, BER/SSBR is considered a major pathway for resolving oxidative DNA lesions (Leandro et al. 2015). Studies in neurodegenerative contexts further suggest that oxidized DNA damage may accumulate beyond the capacity of repair systems, thereby increasing neuronal vulnerability and functional decline (Li et al. 2022; Behrouzi et al. 2022). BER/SSBR proceeds through a coordinated sequence of lesion recognition, damaged base excision when applicable, end processing, gap filling, and ligation. This multistep pathway involves proteins such as PARP1, UDG, APE1, POLB, LIG1, FEN1, and XRCC1. XRCC1 is particularly important because it functions as a scaffold that helps organize and stabilize BER/SSBR complexes, thereby supporting efficient repair completion rather than fragmented or incomplete processing events (Lu et al. 2021).

When XRCC1 function is impaired, the consequences can be especially severe in neural tissue. In humans, loss‐of‐function variants in XRCC1 have been associated with neurological phenotypes, and experimental studies indicate that neuron‐specific XRCC1 deficiency disrupts hippocampal function (McNeill et al. 2011). This is biologically plausible because post‐mitotic neurons cannot dilute DNA lesions through cell division and therefore depend heavily on high‐fidelity repair systems to maintain long‐term viability (Hoch et al. 2017).

The potential contribution of miRNAs to age‐related decline in BER/SSBR remains an important but still incompletely resolved area. At present, many proposed miRNA‐target interactions in DNA repair should be interpreted cautiously unless they are supported by direct binding assays or functional perturbation studies in relevant neural systems. Computational predictions and selected observational studies have nevertheless suggested potentially meaningful links (Turko et al. 2025). For example, miR‐34a, which has frequently been reported to increase with age in the heart and brain, has been predicted to target XRCC1, FEN1, and UDG. Likewise, miR‐181 has been proposed to regulate PARP1, and miR‐146b has been suggested as a candidate regulator of XRCC1 (Xia et al. 2017). These relationships should be distinguished by evidence level, since some are currently based on prediction or expression association rather than reporter validation, Argonaute‐supported binding evidence, or functional knockdown and overexpression studies. The hypothesis that age‐associated miRNA shifts progressively weaken neuronal DNA repair remains mechanistically attractive, but it still requires stronger validation in cell type‐specific and brain‐relevant experimental models, particularly in terminally differentiated neurons (Lee et al. 2017).

## 5 Nutrient‐Sensing Drift in Aging: MiRNAs Around the IGF‐1/Insulin Axis

Deregulated nutrient sensing is widely recognized as a central feature of aging. The IGF‐1/insulin pathway, together with downstream effectors such as AKT, mTOR, and FOXO, constitutes a conserved signaling network that shapes lifespan, stress adaptation, and metabolic homeostasis across species (Turko et al. 2025). Because this axis integrates growth, survival, and energy‐related signals, age‐associated instability within the pathway can influence multiple hallmarks at the same time, including proteostasis, mitochondrial function, cellular senescence, and tissue repair capacity (Martins et al. 2016).

Several miRNAs have been implicated in age‐related changes within this signaling network, although the strength of evidence and tissue specificity vary substantially. In skeletal muscle, miR‐126 has been associated with altered muscle plasticity and has been reported to suppress components of IGF‐1 signaling, raising the possibility that increased miR‐126 may contribute to reduced anabolic or reparative signaling in aging tissue (Baht et al. 2020). miR‐190b has likewise been linked to reduced IGF‐1 levels and to insulin resistance‐related phenotypes, although the available data remain more suggestive than definitive, and direct causal evidence in brain aging is still limited (Xu et al. 2018).

miR‐1 is often discussed as a regulator of IGF‐1‐related signaling in both cardiac and skeletal muscle, and other miRNAs, including miR‐206 and miR‐320, have also been reported to influence components of this pathway (Yoshida and Delafontaine 2020). These findings support the broader idea that relatively small shifts in miRNA expression may have amplified downstream consequences because AKT, mTOR, and FOXO signaling are highly sensitive to upstream regulatory tone. At the same time, many of these observations come from peripheral tissues, so their relevance to brain aging should be interpreted with caution unless supported by neural or brain‐region‐specific data (Qiang et al. 2025).

Nutrient sensing is not governed solely by IGF‐1 abundance itself. PTEN acts as a major negative regulator of PI3K/AKT signaling, meaning that miRNAs that suppress PTEN can shift pathway activity upward rather than downward. In this context, miR‐216a, miR‐217, and miR‐21 have all been described as PTEN‐targeting miRNAs (Qu et al. 2019). However, simply listing PTEN‐targeting miRNAs is not sufficient mechanistically. Their significance in aging depends on whether they are increased or decreased in the relevant aging context, since opposite expression changes would be expected to produce opposite effects on PI3K/AKT pathway activity. For miR‐21, elevated expression has been reported in several aging‐related and inflammatory settings, whereas the age‐dependent direction of change for miR‐216a and miR‐217 appears more context‐dependent and is not yet consistently defined in healthy brain aging (Turko et al. 2025). This point is important because apparent contradictions across studies may reflect species differences, tissue specificity, or comparisons between physiological aging and disease‐associated remodeling rather than healthy aging alone.

SIRT1 is another key node within this network and has long been linked to longevity, metabolic adaptation, and stress resistance (McBurney et al. 2013). In endothelial cells, miR‐217 tends to increase with age and has been shown to target SIRT1, potentially contributing to reduced stress resilience and metabolic dysfunction. miR‐34a has also been reported to repress SIRT1, and this interaction is especially relevant because it can participate in the p53/miR‐34a/SIRT1 regulatory circuit, which links stress responses, senescence‐associated signaling, and metabolic control (Wang et al. 2021). This loop is mechanistically important because increased p53 activity can induce miR‐34a, which in turn suppresses SIRT1, thereby further reinforcing p53‐related signaling. In aging tissues, such feedback may promote a shift away from repair and adaptive resilience and toward persistent stress signaling (Yamakuchi and Lowenstein 2009).

Taken at the level of pathway architecture, the available evidence suggests that miRNAs may fine‐tune nutrient‐sensing networks rather than simply silence them. What remains unresolved is which of these interactions are directly validated in brain aging, which are inferred from peripheral systems, and which are still based mainly on prediction or association. These distinctions are essential if miRNA‐related nutrient sensing is to be interpreted rigorously in the context of neurodegeneration and neural recovery (Badi et al. 2015; Blauensteiner et al. 2021) (Table 1).

**TABLE 1:** Core aging hallmarks and candidate points of miRNA action with attention to mechanism and evidence context.

Aging hallmark	Key biological problem in aging	Example miRNAs mentioned	Main targets/pathways	Expected effect	Evidence context	Reference
Genome instability/DNA repair decline	Damage accumulates, repair capacity declines, and neurons cannot dilute lesions by cell division	miR‐34a, miR‐181, miR‐146b	BER/SSBR‐related genes including XRCC1, FEN1, UDG, and PARP1	Lower repair efficiency may increase neuronal vulnerability and contribute to neurodegenerative risk	Several interactions are currently supported mainly by prediction and expression association; direct functional validation remains limited in brain‐relevant systems	(Demin et al. 2021; Ghediri et al. 2025)
Nutrient‐sensing drift	IGF‐1/insulin signaling becomes dysregulated and metabolic stress increases	miR‐126, miR‐190b, miR‐1, miR‐206, miR‐320; PTEN‐targeting miRNAs including miR‐216a, miR‐217, miR‐21; SIRT1‐targeting miRNAs including miR‐217 and miR‐34a	IGF‐1 signaling, AKT/mTOR/FOXO, PTEN‐mediated restraint of PI3K/AKT, SIRT1‐related stress adaptation	Reduced repair and adaptive signaling, altered stress resistance, and impaired tissue maintenance	Evidence is mixed across tissues. Some interactions are supported outside the CNS, whereas age‐dependent direction of change in healthy brain aging remains unclear for several candidates	(Xu et al. 2018; Dong et al. 2018)
Proteostasis loss	Misfolded and aggregated proteins accumulate	miR‐320, miR‐106a, miR‐26b, miR‐301b, miR‐34a, miR‐146a	Chaperone networks including HSP family members; proteostasis and autophagy‐linked stress responses	Increased proteotoxic stress may favor Alzheimer‐like and Parkinson‐like pathology	For several chaperone‐related interactions, the evidence remains partly predictive rather than uniformly functionally established	(Prodromou et al. 2023; Tang et al. 2025)
Autophagy impairment	Autophagic flux becomes inefficient across initiation, maturation, and trafficking steps	miR‐20a, miR‐106b, miR‐25, miR‐885‐3p, miR‐30a/b, miR‐376b, miR‐216a, miR‐17‐5p, miR‐519a, miR‐630, miR‐374a, miR‐195, miR‐101	ULK1/2, Beclin‐1, UVRAG, ATG14, RAB5A, ATG2, ATG9, ATG18	Reduced clearance promotes abnormal protein and organelle accumulation and may worsen neurodegeneration and recovery potential	This is one of the more densely populated areas of miRNA literature, but evidence strength still differs substantially across individual miRNA‐target pairs	(Birgisdottir et al. 2019; Anwar et al. 2019)
Mitochondrial dysfunction	Electron transport and redox balance decline, while reactive oxygen species increase	miR‐34a, miR‐146a	Electron transport chain genes and oxidative phosphorylatio*n*‐related proteins	Reduced ATP generation and increased oxidative stress may promote synaptic dysfunction and neuronal injury	Associative evidence is relatively common, but direct causal mapping of brain‐specific targets remains incomplete	(Kuzmiak‐Glancy et al. 2022; Bayliak et al. 2023)
Stem cell exhaustion	Self‐renewal capacity declines, while senescence and differentiation pressures increase	let‐7, miR‐145, miR‐499, miR‐371, miR‐369, miR‐34a/b/c, miR‐720	HMGA2, p16 / p19 axis, OCT4, SOX2, KLF4, NANOG, N‐MYC	Reduced regenerative capacity weakens tissue repair and resilience with age	Much of this evidence comes from stem or progenitor systems; extrapolation to adult brain repair should be made carefully	(Villodre et al. 2019; Grubelnik et al. 2020)
Telomere attrition	Telomere maintenance and end protection become compromised, increasing senescence risk	miR‐138, miR‐155, miR‐34a	TERT, TERF1, TERF2, PNUTS	Telomere dysfunction may promote senescence, fibrosis, and cell loss in vulnerable tissues	These interactions should be interpreted within telomere maintenance biology rather than as a generic DNA repair pathway	(Vilkeviciute et al. 2022; Xia et al. 2020)
Epigenetic alteration	DNA methylation, chromatin regulation, and transcriptional programs shift with age	miR‐29 family, miR‐146a, miR‐34a	DNMT3A, DNMT3B, and HDAC‐related regulatory nodes	Altered brain gene‐expression programs may contribute to reduced plasticity and increased inflammatory tone	Some proposed targets remain predictive, so direct evidence should be distinguished from inferred regulatory links	(Chamani et al. 2016; Payet et al. 2021)
Cellular senescence and SASP	Senescence‐associated secretory signaling amplifies inflammation and tissue dysfunction	miR‐34a, miR‐146a	p53, NF‐κB, STAT3‐associated regulatory logic	Persistent stress signaling may accelerate tissue dysfunction and neurodegenerative vulnerability	Mechanistic support is stronger at the pathway level than for every individual direct target assignment	(Zeng et al. 2022; Raucci et al. 2021)
Altered intercellular communication	Circulating proteins and extracellular vesicle‐associated signals shift with age	Exosomal miRNAs rather than one single dominant miRNA	Young versus old plasma effects; candidate mediators include GDF11 and CCL11	Systemic signaling can enhance or suppress plasticity, cognition, perfusion, and tissue recovery	Evidence supports systemic influence on aging phenotypes, but the dominant mediators and their tissue sources remain incompletely resolved	(Yu et al. 2021; Fu et al. 2018)

## 6 MicroRNAs Can Disrupt Protein Quality Control

Proteostasis refers to the integrated cellular systems that preserve protein folding, stabilize conformational integrity, and remove damaged or misfolded proteins through degradation pathways such as the ubiquitin‐proteasome system and the lysosomal‐autophagy network (Shukla and Narayan 2025). During aging, this balance progressively deteriorates, and the resulting loss of protein quality control is thought to become even more pronounced in age‐related disorders. Persistent accumulation of unfolded, misfolded, or aggregation‐prone proteins is closely linked to neurodegenerative diseases, including Alzheimer's disease and Parkinson's disease (Sharma et al. 2024). In the aging brain, proteostasis failure is especially consequential because neurons are long‐lived, highly specialized cells with limited replacement capacity and sustained exposure to metabolic and oxidative stress.

A major component of the proteostasis network is the molecular chaperone system, which assists protein folding, limits inappropriate intermolecular interactions, and helps prevent the accumulation of toxic conformers. This chaperone network functions as a critical buffer against proteotoxic stress and has clear relevance to neurodegenerative vulnerability (Sharma et al. 2024). Major chaperone families include HSP90, HSP70, HSP40, HSP110, and related co‐chaperones, each contributing to distinct aspects of folding surveillance, refolding, or degradation triage. Because these proteins help determine whether damaged proteins are refolded, sequestered, or degraded, miRNA‐mediated changes in chaperone expression could shift the cellular response away from adaptation and toward proteotoxic collapse (Roy and Tamuli 2022). Several observations suggest that miRNAs may influence this balance. miR‐320 has been reported to increase during cardiac injury and to affect HSP20‐associated protective pathways, although the relevance of this interaction to brain aging or neurodegeneration remains indirect unless supported by neural data (Liu et al. 2016). Likewise, elevated levels of miR‐106a, miR‐26b, and miR‐301b have been linked to disruption of HSP70‐related autophagic control, a mechanism that may promote α‐synuclein‐associated pathology in some experimental settings (Liu et al. 2016). These findings are mechanistically interesting, but they should be interpreted according to evidence level, since some studies provide functional data whereas others rely more heavily on expression association or predicted targeting.

**FIGURE 1:**
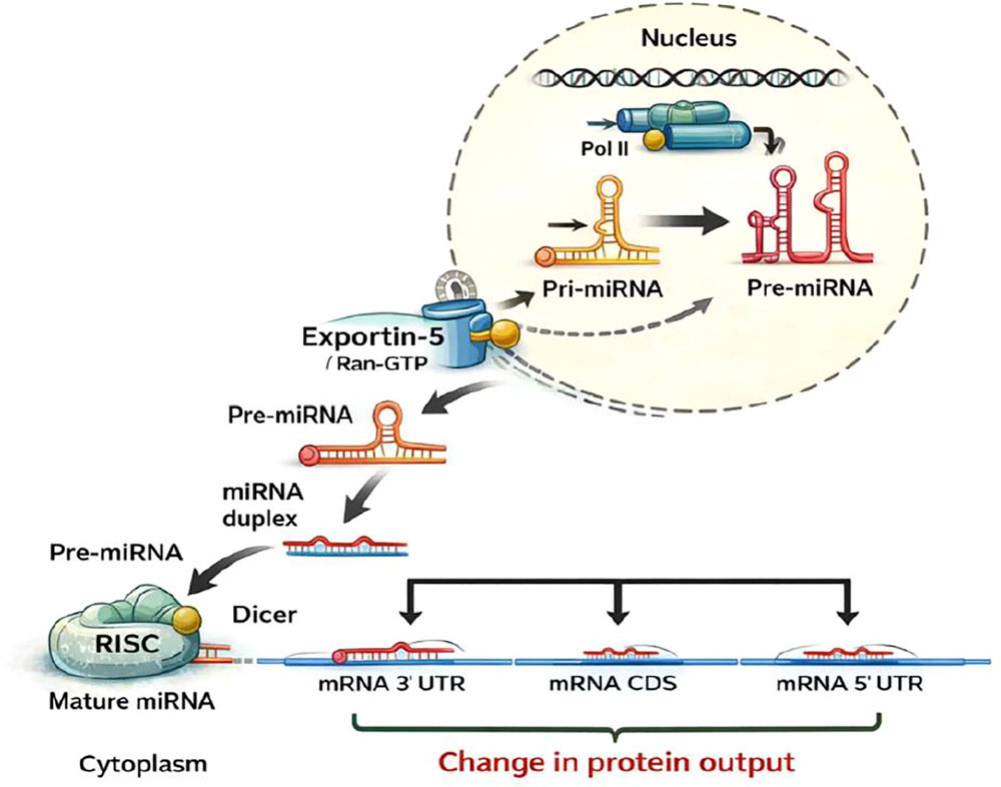
Canonical miRNA biogenesis and gene‐silencing output relevant to aging and neurodegeneration. miRNAs are transcribed mainly by RNA polymerase II as primary transcripts in the nucleus, processed into precursor miRNAs, exported to the cytoplasm by Exportin‐5/Ran‐GTP, and further cleaved by Dicer to generate a miRNA duplex. The mature miRNA is then incorporated into RISC and guides the complex to target mRNAs at the 3′ UTR, coding region, or 5′ UTR, resulting in translational repression and altered protein output. These regulatory effects are relevant to pathways involved in aging and neurodegenerative vulnerability.

In aging cardiac tissue and in the brains of patients with Alzheimer's disease, miR‐34a and miR‐146a are frequently reported to be elevated, although the exact pattern varies across cohorts, tissues, and disease stages. Computational and candidate‐targeting studies have suggested that miR‐34a may regulate HSP20, HSP40, and HSP72, whereas miR‐146a has been proposed as a regulator of HSP90 (Maldonado‐Lasuncion et al. 2019). At present, many of these chaperone‐related miRNA interactions should be described cautiously unless they are supported by direct reporter assays, Argonaute‐associated binding evidence, or perturbation studies in disease‐relevant neural cells. Even so, the available literature supports the idea that miRNA dysregulation may weaken protein quality‐control capacity and thereby intensify proteotoxic stress in aging and neurodegeneration (Frédérick et al. 2024) (Figure 1).

## 7 MicroRNAs in Autophagy Control and Cargo Clearance

Proteostasis depends not only on molecular chaperones but also on efficient autophagic clearance of damaged proteins and dysfunctional organelles. Autophagy is therefore a major determinant of intracellular quality control, particularly in long‐lived cells such as neurons. Recent studies indicate that miRNAs participate in the regulation of both autophagy initiation and later maturation steps, suggesting that they may influence the efficiency of the entire autophagic flux rather than a single checkpoint (Munoz‐Braceras and Escalante 2016; Lange et al. 2025). This point is especially important in brain aging, where even partial slowing of autophagic turnover may favor the accumulation of toxic proteins and defective mitochondria over time.

Autophagy initiation begins with activation of the ULK complex, which includes ULK1 or ULK2 together with FIP200, ATG13, and ATG101. Several miRNAs have been reported to regulate this step. miR‐20a and miR‐106b can directly suppress ULK1, implying that increased expression of these miRNAs may reduce autophagy initiation (Hama et al. 2025). In contrast, inhibition of miR‐25 has been shown to increase ULK1 expression in some systems and, under certain conditions, promote autophagy‐associated cell death. ULK2 has also been identified as a direct target of miR‐885‐3p, suggesting that distinct regulatory effects may occur depending on whether ULK1‐ or ULK2‐dependent initiation is being examined (Hama et al. 2025). These observations illustrate that miRNA effects on autophagy are highly context dependent and cannot be interpreted as uniformly protective or uniformly harmful. A second regulatory layer involves nucleation of the isolation membrane. Several miRNAs, including miR‐30a/b, miR‐376b, miR‐216a, and miR‐17‐5p, have been reported to suppress Beclin‐1, thereby reducing early autophagosome formation (Gao 2025). Beclin‐1 may also be targeted by miR‐519a. In parallel, miR‐630 and miR‐374a can inhibit UVRAG, a factor that interacts with Beclin‐1 and promotes autophagic activity. ATG14, a key component of the class III PI3K‐Beclin‐1 complex, has also been reported as a target of miR‐195, which may further limit nucleation‐related membrane assembly (Wu et al. 2019; Eslami et al. 2019). Because Beclin‐1 and its associated complexes occupy a central position in autophagy regulation, miRNA‐mediated suppression at this level may have broad downstream effects on cargo handling and cellular stress adaptation.

**FIGURE 2:**
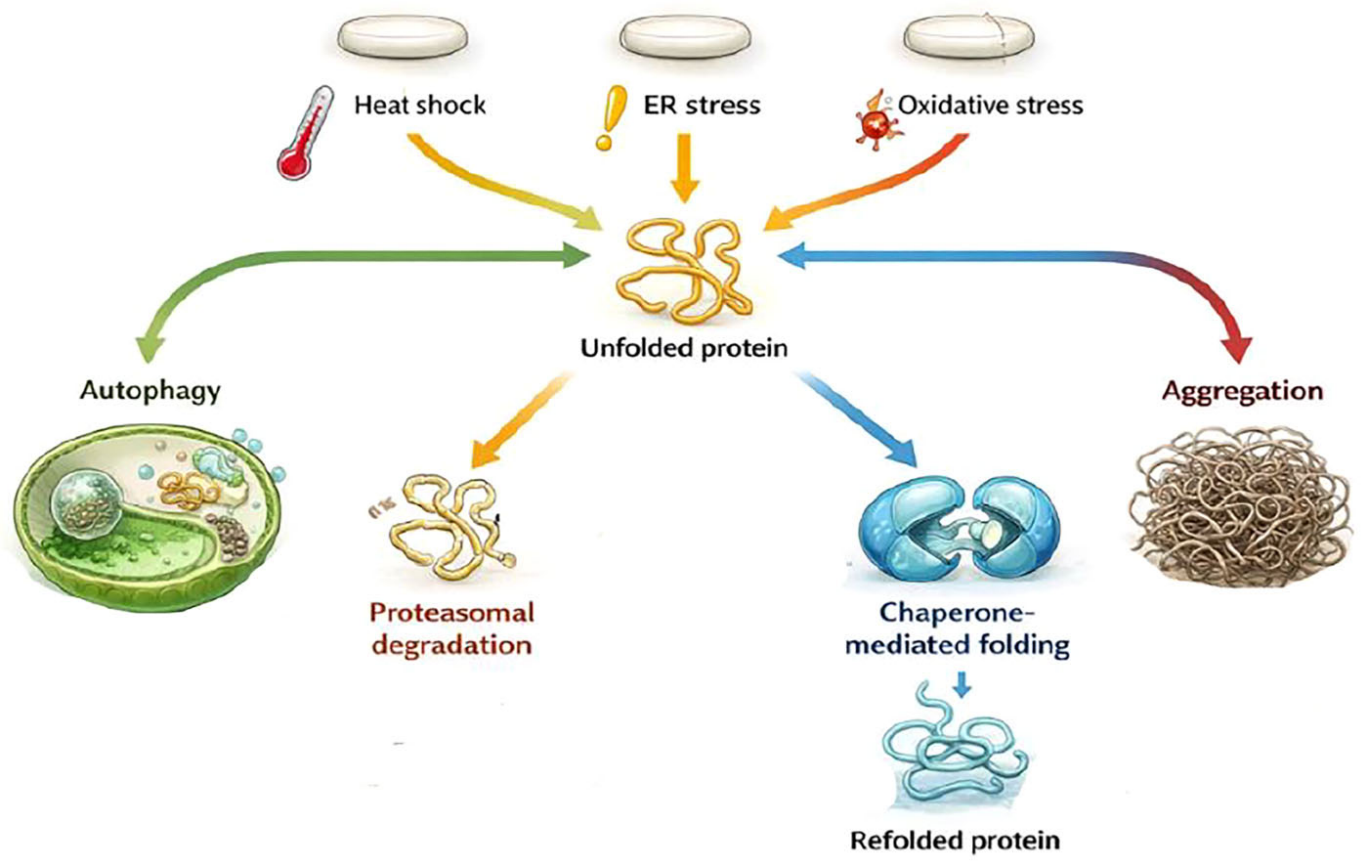
Proteostasis decision points under cellular stress in aging and neurodegeneration. Cellular stressors such as heat shock, endoplasmic reticulum stress, and oxidative stress increase the accumulation of unfolded proteins. These proteins can be refolded through chaperone‐mediated mechanisms or removed through proteasomal degradation and autophagic clearance. If proteostasis capacity is impaired, protein aggregation may occur, thereby contributing to neuronal dysfunction and degeneration. miRNAs can influence this balance by regulating components of the cellular protein quality‐control network.

RAB5A, a small GTPase involved in vesicular trafficking, can support autophagosome formation through interactions with hVPS34 and Beclin‐1, thereby contributing to the membrane platform required for autophagic vesicle development. miR‐101 has been reported to target RAB5A and to inhibit autophagy, suggesting that this miRNA may also affect the vesicular assembly stage of the pathway (Sawa‐Makarska et al. 2020). Later phases of autophagy are likewise subject to miRNA regulation through targets such as ATG2, ATG9, UVRAG, and ATG18, indicating that defects may arise not only during initiation but also during autophagosome maturation, trafficking, and fusion with lysosomes (Sawa‐Makarska et al. 2020). For this reason, reduced autophagic efficiency in aging should not be viewed only as a failure to start the pathway; it may also reflect impaired completion of flux, which is especially relevant when interpreting miRNA effects in neurodegenerative models (Figure 2).

## 8 MicroRNAs and Mitochondrial Energy Defects During Aging

Mitochondrial dysfunction is a well‐established feature of aging in both the brain and the heart, two organs with especially high and continuous energy demands. Transcriptomic profiling across human aging cohorts has suggested that expression of multiple mitochondrial genes declines during midlife‐to‐late‐life transitions, although the precise timing varies across datasets, brain regions, and analytical platforms and should not be reduced to a single universal age cutoff (Cimbalo et al. 2021). Among the affected genes are those involved in ATP synthesis, electron transport, and cytochrome c oxidase‐associated respiratory activity, all of which are essential for sustained oxidative phosphorylation.

Defects in the mitochondrial electron transport chain have been repeatedly linked to late‐onset Alzheimer's disease and related neurodegenerative states. One commonly reported abnormality is reduced cytochrome c oxidase activity, which can contribute to impaired ATP production, increased reactive oxygen species generation, and broader metabolic instability (Misrani et al. 2021). In neurons, these changes are particularly damaging because synaptic function, axonal transport, ion homeostasis, and stress adaptation all depend on reliable mitochondrial output (Li et al. 2020).

A number of miRNAs have been reported to increase in Alzheimer's‐related settings, but miR‐34a and miR‐146a are among the most frequently discussed because they have been linked to mitochondrial and electron transport‐related targets (Li et al. 2020). Here again, the level of support differs across studies, and it is important to distinguish between differential expression in diseased tissue, computational target prediction, and direct functional evidence in neuronal models. Reduced expression of electron transport‐associated proteins has been observed in Alzheimer's disease brain tissue, and experimental overexpression of miR‐34a has been shown to reduce oxidative phosphorylation and suppress key electron transport‐chain components in neuronal systems (Sarkar et al. 2016). This makes miR‐34a one of the more mechanistically plausible candidates linking age‐related miRNA dysregulation to mitochondrial decline, although the magnitude and direction of its effects are still likely to depend on cell type, disease stage, and local stress conditions.

miR‐146a has also attracted attention because of its broader relationship to inflammatory signaling, which is relevant to mitochondrial biology in aging and neurodegeneration. This is important because mitochondrial dysfunction and neuroinflammation often reinforce one another: impaired respiration can increase oxidative and inflammatory stress, while inflammatory signaling can further damage mitochondrial homeostasis (Catanesi et al. 2026). In this setting, miRNAs that influence both immune pathways and mitochondrial function may contribute to a self‐reinforcing pattern of neuronal vulnerability. Continued work will be needed to determine which mitochondrial miRNA interactions are directly causal in brain aging and which are better understood as secondary responses to degenerative stress (Li et al. 2023).

## 9 MicroRNAs May Contribute to Stem Cell Exhaustion

Genes that maintain stem cell identity regulate self‐renewal, proliferative capacity, lineage commitment, and quiescence, and these programs often become progressively destabilized with age. In this setting, miRNAs have emerged as potential regulators of stem cell exhaustion because they can repress pluripotency‐associated genes while promoting senescence‐linked pathways (Divisato et al. 2020). HMGA2 is one such factor that supports stem‐like properties, and let‐7 is a well‐established regulator of HMGA2. Increased let‐7 activity has been associated with reduced self‐renewal potential and with activation of p16 and p19‐related signaling, suggesting that this axis may accelerate stem cell aging under conditions of chronic stress or repeated proliferative demand (Ancel et al. 2021).

miR‐145 has also been implicated in this process because it can suppress core pluripotency regulators such as OCT4, SOX2, and KLF4, thereby shifting cells away from self‐renewal and toward differentiation. In mesenchymal stem cells, several differentially expressed miRNAs, including let‐7, miR‐499, miR‐371, and miR‐369, have been linked to replicative senescence and reduced stemness (Barta et al. 2016). The miR‐34 family, including miR‐34a, miR‐34b, and miR‐34c, can further inhibit somatic cell reprogramming by repressing NANOG, SOX2, and N‐MYC, while miR‐720 has also been reported to promote differentiation at least in part through NANOG suppression (Zhang et al. 2021). These findings support the idea that miRNAs may help shift aging stem cell systems away from renewal and toward differentiation or senescence by persistently repressing pluripotency‐associated transcriptional programs.

In the context of aging and neurodegeneration, this issue is most relevant when considering adult neural stem and progenitor cell populations rather than pluripotent stem cell models alone. Although many mechanistic studies have been performed in embryonic, induced pluripotent, or mesenchymal stem cell systems, the broader principle may still be informative for brain aging, where reduced regenerative reserve and altered progenitor behavior could limit tissue resilience and recovery capacity (Wang et al. 2016). At the same time, direct evidence linking individual miRNAs to stem cell exhaustion in the aging human brain remains more limited than in non‐neural model systems, so extrapolation should be made cautiously.

## 10 Specific miRNAs May Affect Telomere Maintenance Regulators

In both humans and mice, aging is commonly accompanied by progressive telomere shortening. Telomere length is not entirely passive; however, it is shaped by regulatory systems that preserve chromosome‐end stability. Shortened telomeres are closely associated with cellular senescence and tissue dysfunction, whereas abnormally sustained telomere maintenance can contribute to malignant transformation in certain settings (Calado and Dumitriu 2013). Telomere biology should therefore be discussed in terms of telomere maintenance and end protection rather than as a generic DNA repair pathway. This machinery includes telomerase reverse transcriptase (TERT), the telomerase RNA component (TERC), shelterin‐associated proteins such as TERF1 and TERF2, and additional telomere‐associated regulators including dyskerin‐related factors (Yamauchi et al. 2025; Kumar et al. 2018). In this context, several miRNAs have been linked to telomere‐associated genes and may therefore influence the pace of telomere erosion.

Some studies have shown that miR‐138 can target TERT transcripts in thyroid cancer cells, potentially reducing telomerase activity. miR‐155 has been reported to regulate TERF1, which may alter telomere protection dynamics, and miR‐34a overexpression has been associated with shorter telomeres in gallbladder cancer tissues while also intersecting with apoptosis and senescence‐related pathways (Ullah and Sun 2019; Leão et al. 2018). These observations are mechanistically relevant, but much of the evidence comes from cancer models, where telomere regulation is strongly shaped by proliferation and oncogenic stress. For that reason, these interactions should not automatically be interpreted as established mechanisms in healthy brain aging or normal neural tissue.

Among the candidate miRNAs discussed in age‐related telomere biology, miR‐34a has attracted particular attention in the aging heart. Its expression appears to increase with age in both human and mouse cardiac tissue, and experimental studies suggest that elevated miR‐34a may contribute to telomere attrition, cardiomyocyte death, and impaired myocardial performance (de Lucia et al. 2017). One proposed target is PNUTS, also known as PPP1R10, a factor that has been linked to TERF2‐associated telomere regulation in some mechanistic models (Xia et al. 2020). In vivo inhibition of miR‐34a using antagomiR‐based approaches has been reported to restore PNUTS expression, reduce cell death and fibrosis after myocardial infarction, and improve functional recovery. Computational analyses have also suggested that miR‐34a may target TERT and TERF2, although these predicted interactions require stronger direct validation before they are described as confirmed regulatory events (Bernardo et al. 2022).

The broader significance of these data is that miRNA dysregulation may influence telomere maintenance through multiple nodes, including telomerase activity, shelterin‐associated protection, and senescence‐related signaling. What remains less certain is which of these relationships operate directly in brain aging, which are tissue‐specific, and which are primarily supported by associative or predictive evidence rather than direct functional testing (Williams et al. 2017). In age‐related cardiovascular tissues, telomere attrition has been linked to senescence in cardiomyocytes, endothelial cells, and vascular smooth muscle cells, providing a plausible route by which miRNA‐associated telomere dysregulation could contribute to organ decline (Xia et al. 2020).

## 11 miRNAs and Shifts in Gene‐Expression Control During Aging

The genome encodes the molecular components required to build and maintain tissues, but the biological output of these components depends on how gene expression is regulated across time and cellular context (Misteli 2020). During aging, progressive alterations in transcriptional and epigenetic control help shape the aging phenotype and may contribute to organ‐specific pathology, particularly in the brain, where neuronal maintenance, glial regulation, synaptic adaptability, and neurovascular stability depend on tightly coordinated gene‐expression programs (Krauss and de Haan 2016). Comparative profiling studies in brain regions such as the cortex and cerebellum further suggest that age‐associated miRNA changes are linked to genes involved in cognitive decline and neurodegenerative vulnerability. Rather than being driven by a single upstream defect, these regulatory shifts likely emerge from multiple interacting layers, including altered DNA methylation, histone modifications, changes in transcriptional machinery, chromatin‐state remodeling, and disturbances in nucleocytoplasmic trafficking (Singh and Paramanik 2024). This layered model is especially relevant to brain aging because it helps explain why relatively modest changes in miRNA abundance can become amplified across plasticity, metabolic, inflammatory, and repair‐related networks (Tregub et al. 2023) (Table 2).

**TABLE 2:** Brain‐aging transcriptional signatures and candidate miRNA‐linked regulatory directions.

Brain aging observation	Direction with age	Example functional gene groups	How miRNAs may contribute	Possible consequence for neurodegeneration or recovery	Evidence context	Reference
Maintenance and adaptive programs weaken	Mostly down	Gray and white matter plasticity genes, mitochondrial genes, learning and memory support, intracellular transport, oxidative stress defense, DNA repair, transcriptional and epigenetic regulators	miRNAs may repress translation of plasticity‐, repair‐, and metabolism‐related genes; miR‐34a is repeatedly implicated across several of these domains	Reduced circuit resilience, impaired learning and memory, and lower recovery capacity after injury or stress	MiR‐34a appears recurrently in prediction‐based and associative studies, but direct validation differs across individual targets and brain regions	(Michely et al. 2017; Andolina et al. 2021)
Immune and inflammatory programs increase	Mostly up	Innate immune signaling, cytokine‐related pathways, glial activation programs	miRNAs can modulate TLR‐NF‐κB signaling and may either restrain or sustain inflammatory activity depending on context and target repertoire	Chronic neuroinflammation may intensify tau‐related changes, oxidative and nitrosative stress, and neuronal injury	Pathway‐level support is substantial, but the direction and impact of individual miRNAs remain context dependent	(Hanley et al. 2024; Ureña‐Peralta et al. 2020)
Epigenetic drift contributes to transcriptomic remodeling	Mixed but progressive	DNA methylation changes, histone modifications, non‐coding RNA shifts	miR‐29 family can regulate DNMTs; miR‐146a has been proposed to influence DNMT3A; miR‐34a and miR‐146a have been linked to HDAC‐related regulation	Persistent reprogramming of gene output may promote long‐term decline in plasticity and metabolic adaptation	Some interactions are supported by direct evidence in non‐neural systems, whereas others remain predictive in the context of brain aging	(Payet et al. 2021; Aschner et al. 2024)
Activity‐dependent gene regulation becomes fragile	Functional downshift	Immediate early genes, promoter‐associated repair machinery, NPAS4‐dependent plasticity programs	miR‐34a has been proposed to target TOP2, TDP2, NPAS4, and additional plasticity‐related genes	Reduced synaptic remodeling and poorer adaptive responses may contribute to cognitive decline and limited neurorestoration	Several of these relationships are still prediction based and require stronger functional testing in neural systems	(Sarkar et al. 2019; Xu et al. 2018)
White matter adaptation declines	Functional downshift	Myelination regulators such as MYRF, motor‐learning‐associated programs	miR‐34a has been predicted to target MYRF and thereby influence myelin‐related transcriptional control	Reduced white matter plasticity may impair processing efficiency, learning, and post‐injury adaptation	This is a plausible mechanistic link, but direct experimental confirmation remains limited	(Li et al. 2025; Zhang et al. 2021)

### 11.1 miRNAs and DNA Methylation Changes

miRNAs can influence DNA methylation patterns, and in some contexts this contributes to aberrant methylation states. Members of the miR‐29 family are well known for targeting DNA methyltransferases such as DNMT3A and DNMT3B in several non‐neural systems, thereby reducing methylation capacity at specific loci (Sun et al. 2020). This point is mechanistically important because it shows that miRNAs can act upstream of broader epigenetic remodeling rather than only fine‐tuning isolated transcripts. In the nervous system, conditional loss of DNMT1 and DNMT3A in forebrain excitatory neurons has been shown to impair long‐term synaptic plasticity in the hippocampal CA1 region and to disrupt learning and memory, indicating that methylation control remains essential for mature neuronal function (Sun et al. 2020). Computational analyses have also suggested that miR‐146a may target DNMT3A, and miR‐146a expression has been reported to increase in late‐onset Alzheimer's disease relative to age‐matched controls (Maffioletti et al. 2019). At present, however, the proposed miR‐146a‐DNMT3A connection in the aging brain should be described cautiously unless it is supported by direct binding or perturbation‐based validation in neural cells (Morita et al. 2013).

### 11.2 miRNAs and Histone Regulation

Histone acetylation is strongly linked to learning, memory, and synaptic plasticity in animal models. Much of the literature has focused on class I histone deacetylases, particularly HDAC2, because reducing HDAC2 activity in the brain has been associated with improved memory formation and enhanced synaptic plasticity (González et al. 2020). By contrast, class IIa HDACs such as HDAC4, HDAC5, HDAC7, and HDAC9 remain less completely understood, even though they also act as transcriptional repressors and can shuttle between the nucleus and cytoplasm in response to signaling cues. Selective loss of HDAC4 in the brain has been linked to impairments in hippocampus‐dependent learning, memory, and long‐term plasticity, underscoring the fact that histone regulators do not all operate in the same direction (González et al. 2020). This distinction matters because age‐related miRNA effects on histone regulators may be beneficial, neutral, or detrimental depending on which HDAC is being affected and in which cellular context. Computational studies have suggested that miR‐34a and miR‐146a may target HDAC2, and because both miRNAs are often elevated in Alzheimer's disease‐related settings, this interaction has attracted mechanistic interest (Dai et al. 2024). Still, predicted targeting should not be treated as equivalent to experimentally verified repression in aging neurons or glia (Guan et al. 2009).

### 11.3 miRNAs, Transcriptional Machinery, and Plasticity‐Related Genes

Topoisomerase II (TOP2) is an important component of transcriptional regulation because it relieves torsional stress in chromosomal DNA during active transcription. In some neuronal contexts, TOP2 activity is coupled to transient double‐strand break formation at promoters of immediate early genes, after which repair factors such as TDP2 help restore DNA integrity (Al Mahmud et al. 2020). This process has been linked to rapid activity‐dependent transcription and is relevant to synaptic plasticity and memory encoding. Computational analyses suggest that miR‐34a may target TOP2, TDP2, and several immediate early or plasticity‐related genes, including NPAS4 (Sun and Lin 2016). Because NPAS4 is a major regulator of excitatory‐inhibitory balance, activity‐dependent circuit remodeling, and memory formation, suppression of this pathway could have broad consequences for brain aging if it is confirmed experimentally. However, many of these proposed miR‐34a interactions remain predictive or associative rather than directly validated in aging brain tissue or disease‐relevant neuronal models (Sun and Lin 2016).

Beyond immediate early genes, several synaptic plasticity and memory‐related genes that decline in the aging human prefrontal cortex have also been proposed as miR‐34a targets. This may help explain why elevated miR‐34a expression has been associated in some studies with reduced protein output from plasticity‐related pathways. Computational work has further suggested that miR‐34a may target MYRF, a transcription factor that functions as a master regulator of myelination in the central nervous system (Qi et al. 2024). In mice, deletion of Myrf disrupts acquisition of complex motor learning tasks, implying that reduced MYRF expression could impair white matter adaptability (Attia et al. 2019). These observations support the view that miR‐34a may influence both gray matter synaptic plasticity and white matter remodeling, although the magnitude and biological relevance of these effects likely vary across brain region, cell type, and disease stage.

### 11.4 miRNAs and Inflammatory Signaling Pathways

Inflammation is an evolutionarily conserved defense system that protects tissues from infection and injury. Tissue‐resident innate immune cells use pattern‐recognition receptors to detect pathogen‐associated molecular patterns derived from microbes and damage‐associated molecular patterns released from stressed, necrotic, or senescent host cells. Among these receptors, toll‐like receptors are especially important because their activation can trigger NF‐κB, MAPK, and interferon‐related signaling cascades that amplify inflammatory gene expression (Kumar 2022; Jang et al. 2020). In the brain, this signaling network is relevant not only to host defense but also to age‐associated neuroinflammation, where activated glia and stressed neurons produce cytokines and reactive mediators that can influence tau phosphorylation, oxidative stress, and neuronal survival (Sadallah et al. 2015; Chou et al. 2020). Inflammatory signaling is not uniformly harmful, since controlled immune activation can support debris clearance and tissue remodeling after injury. In some experimental Alzheimer‐like models, for example, hippocampal lipopolysaccharide exposure has been reported to transiently enhance clearance of diffuse amyloid‐β deposits. Yet sustained or excessive inflammatory activation can also damage surrounding neural tissue, especially when oxidative and nitrosative stress remain elevated for prolonged periods (Sadallah et al. 2015). For this reason, neuroinflammation in aging and Alzheimer's disease is best viewed as a context‐dependent process that can become maladaptive when regulatory brakes fail or when inflammatory signaling becomes chronically self‐sustaining.

### 11.5 miRNAs May Help Restrain, Reshape, or Prolong Inflammatory Signaling

The immune system must eliminate pathogens and clear damaged tissue while limiting unnecessary injury to healthy cells. A growing body of work suggests that several miRNAs participate in this balancing process (Riaz et al. 2025). Inflammation‐responsive miRNAs such as miR‐21, miR‐146a, and miR‐155 are closely linked to TLR‐NF‐κB signaling and can be induced during inflammatory activation, where they may either dampen upstream signaling components or, in other contexts, reinforce inflammatory circuits indirectly through secondary targets (Yang et al. 2021; Huang et al. 2023). Their role is therefore not binary. The same miRNA may act as a compensatory brake in one setting and contribute to chronic inflammatory remodeling in another, depending on cell type, disease state, and target availability (Elton et al. 2013).

## 12 How the Blood–Brain Barrier Shapes Inflammatory Signaling

Communication between the central nervous system and the peripheral immune system is now understood to be bidirectional and highly dynamic. Rather than functioning as isolated compartments, the brain and the immune system influence one another across physiological aging, systemic inflammation, and neurodegenerative disease (Riaz et al. 2025). Within this interface, the blood–brain barrier is a central regulator of neuroimmune communication, not a completely sealed wall but a highly selective and biologically active boundary (Erickson and Banks 2019). Compared with peripheral vascular endothelium, brain endothelial cells are distinguished by specialized tight junctions, low paracellular permeability, and close functional interaction with astrocytes, pericytes, and other components of the neurovascular unit (Kadry et al. 2020). Glial‐derived signals help maintain endothelial specialization and barrier integrity, thereby restricting uncontrolled passage of circulating molecules and immune cells into the central nervous system (Smith et al. 2022). This point is especially important in brain aging because the blood–brain barrier does not simply block entry into the CNS. It filters, senses, and responds to systemic signals, meaning that age‐related or disease‐related changes in barrier function can reshape inflammatory signaling within neural tissue.

Clinical and epidemiological studies suggest that peripheral infection or systemic immune activation may be associated with cognitive impairment in adults. Experimental work in animal models likewise indicates that systemic inflammation can increase hippocampal inflammatory signaling and impair behavior. In some settings, it has also been linked to Alzheimer‐like features, including tau hyperphosphorylation, increased Aβ42 accumulation and plaque burden, and deficits in learning and memory (Qi et al. 2017; Ponce‐Lopez 2025). These observations do not imply that infection directly causes Alzheimer's disease, but they do support the view that systemic inflammation can promote pathological changes that resemble or intensify neurodegenerative processes under susceptible conditions.

### 12.1 BBB Breakdown, miRNAs, and Immune‐Cell Entry

Under physiological conditions, tight junctions within the blood–brain barrier help restrict the entry of unwanted cells and circulating factors, including pathogens and inflammatory mediators. However, both systemic inflammation and neuroinflammation can weaken barrier integrity, increasing permeability and facilitating the entry of blood‐derived proteins, monocytes, leukocytes, and, in some cases, infectious agents (Kim et al. 2025). This creates a potentially self‐reinforcing loop in which inflammation disrupts barrier function, barrier dysfunction permits greater immune entry, and the resulting neuroinflammation further destabilizes the barrier (Aborode et al. 2025).

Several miRNAs have been implicated in this process. Circulating cytokines can induce miR‐155 in brain endothelial cells, and increased miR‐155 has been associated with tight‐junction disruption and increased permeability. In a different context, Aβ42 accumulation in Alzheimer‐related settings has been linked to reduced endothelial miR‐107 expression, and restoration of miR‐107 has been reported to improve barrier properties in some experimental models (Pena‐Philippides et al. 2018). Inflammatory astrocyte‐derived signals may also weaken blood–brain barrier stability, while miR‐34a‐associated mitochondrial dysfunction could further compromise endothelial resilience by impairing cellular energy balance and stress adaptation (Aborode et al. 2025). These findings suggest that miRNAs may influence barrier integrity simultaneously through inflammatory signaling, endothelial metabolism, and junctional protein regulation, which is mechanistically more informative than viewing BBB leakage as a purely structural defect.

For circulating immune cells to enter CNS tissue, they must first adhere to the brain endothelium. This step depends on endothelial adhesion molecules such as ICAM‐1 and VCAM‐1. In response to inflammatory cytokines including TNF‐α, IFN‐γ, and IL‐1, brain microvascular endothelial cells can strongly upregulate these adhesion molecules, thereby facilitating leukocyte recruitment and transendothelial migration into the brain parenchyma (Al‐Obaidi and Desa 2018; Nishihara et al. 2020). This mechanism provides a direct link between peripheral cytokine signaling and increased CNS immune‐cell entry. By contrast, overexpression of miR‐98 and let‐7g* in brain microvascular endothelial cells has been shown to reduce monocyte adhesion and decrease barrier permeability, suggesting that some miRNAs may preserve barrier function rather than disrupt it (Al‐Obaidi and Desa 2018; Nishihara et al. 2020). This distinction is important because inflammation‐associated miRNAs should not be treated as uniformly pathogenic. Their effects depend on cell type, target repertoire, and the inflammatory context in which they are induced.

### 12.2 Aging‐Related Changes in BBB Function

Even in the absence of overt neurological disease, aging itself can alter blood–brain barrier function. Older adults and aged animals may show reduced transport of key metabolic substrates, including glucose and choline, from the circulation into the CNS, and such changes may contribute to declining neural performance (Garcia‐Gallardo and Campbell 2025). Aging therefore appears to create a background state of barrier vulnerability characterized by impaired transport efficiency and, in some settings, increased permeability. When superimposed on systemic inflammation or neurodegenerative pathology, this vulnerable state may allow peripheral signals to access the brain more readily and intensify local inflammatory responses.

### 12.3 CNS Regions Without a Fully Classical BBB

Not all regions of the central nervous system are protected by a fully classical blood–brain barrier. Circumventricular organs, ventricular surfaces, the choroid plexus, and the meninges show weaker tight‐junction organization and, in some regions, fenestrated endothelial features that permit more direct communication with circulating blood (Ueno 2017; Bentivoglio et al. 2018). These sites are particularly relevant to neuroimmune signaling because they can function as anatomical interfaces where peripheral inflammatory cues are sampled more readily than in tightly sealed brain regions. For this reason, they may serve as important entry points or sensing zones through which systemic immune activity influences CNS inflammation during aging and disease (Frederick et al. 2022).

## 13 miRNA‐Based Translational Tools: Biomarkers and Therapeutic Possibilities

Across many disease settings, miRNAs have attracted attention as candidate biomarkers, in part because they are relatively stable in biological fluids. In blood, many miRNAs are not present as unprotected molecules. Instead, they are associated with lipoproteins, ribonucleoprotein complexes, or extracellular vesicles such as exosomes, which can shield them from rapid degradation by RNases (Lonnerdal 2019). This biochemical stability is one reason circulating miRNAs are considered attractive for disease detection and monitoring, particularly in disorders where tissue biopsy is difficult or undesirable. At the same time, translating this promise into clinical use remains difficult because disease states are heterogeneous, patient populations are variable, and laboratory workflows are not standardized across centers (Ugolini et al. 2020).

Several pre‐analytical and analytical factors can substantially affect miRNA readouts. Sample timing is especially important, since early‐stage and late‐stage disease, as well as pre‐treatment and post‐treatment states, may generate very different molecular profiles (Kyriakidis et al. 2025). In addition, RNA isolation methods, normalization strategies, and quantification platforms can influence the apparent magnitude or even direction of differential expression. For this reason, circulating miRNA signatures should not be interpreted as disease‐specific by default, because part of the observed variation may reflect technical noise, cohort composition, or treatment‐related effects rather than underlying disease biology alone (Lucca and Sommerville 2018). This issue is particularly important in aging‐related neurological disorders, where systemic inflammation, comorbidities, and concurrent medications can all reshape circulating RNA profiles.

Beyond their biomarker potential, miRNAs are also being explored as therapeutic targets. In principle, if a disease is associated with reproducible overexpression of a pathogenic miRNA, that miRNA could be inhibited using antisense oligonucleotides or anti‐miR strategies. Conversely, if a beneficial miRNA is depleted, synthetic mimics might be used to restore its regulatory function (Song et al. 2022). Although this therapeutic logic is conceptually appealing, it is not straightforward in practice because individual miRNAs usually regulate multiple mRNAs, and each target transcript may also be under the control of several different miRNAs. As a result, altering a single miRNA can reshape multiple pathways simultaneously, creating the possibility of both therapeutic benefit and unintended off‐target effects (Chen and Lu 2018; To et al. 2020).

An additional strategy involves targeting miRNA recognition rather than miRNA abundance itself. In this approach, decoy sequences or competing endogenous RNAs are used to sequester miRNAs away from endogenous target transcripts, thereby relieving repression of selected mRNAs (Verheyden et al. 2024). One example discussed in the literature is Rpph1, which can compete with CDC42 for binding to miR‐326‐3p and miR‐330‐5p. Increased Rpph1 expression has been associated with enhanced CDC42 translation and greater dendritic spine formation, suggesting that manipulation of miRNA availability may influence synaptic structure indirectly (Cai et al. 2017; Bartelt‐Kirbach and Golenhofen 2023). This type of approach is mechanistically interesting for neurorestorative research, but it also requires caution because decoy systems may bind more than one miRNA and can therefore perturb broader regulatory networks than initially intended.

The translational field is further complicated by the fact that many proposed miRNA targets remain incompletely validated. Predicted targeting is not equivalent to direct biological regulation in tissue, and therapeutic design becomes much less reliable when the underlying miRNA‐mRNA interactions have not been confirmed experimentally in disease‐relevant cell types (Li et al. 2025). This limitation is especially important in the central nervous system, where neuronal, glial, endothelial, and immune cells may each respond differently to the same miRNA perturbation (Chen and Lu 2018). Accordingly, delivery route, dose, tissue specificity, and cell‐type selectivity are not secondary technical issues. They are central determinants of whether a miRNA‐based intervention will be effective or harmful.

Another important consideration is treatment context. In age‐related neurological conditions, miRNA profiles should be interpreted alongside ongoing therapeutic regimens because treatment itself can alter gene‐regulatory states (Shamaeizadeh and Mirian 2024). For example, in stroke and other acute brain injuries, reperfusion therapies and other interventions may activate molecular programs related to inflammation, vascular remodeling, and tissue repair. If sampling time is not carefully controlled, treatment‐induced miRNA changes may be misread as disease‐defining signatures (Stucky 2025). The same principle applies to chronic neurodegenerative disorders, where symptomatic or disease‐modifying therapies may also reshape circulating or tissue‐associated miRNA patterns.

Despite these limitations, recurrent miRNA signatures continue to emerge across studies of disease progression, tissue injury, and neurodegeneration. Some of these patterns may ultimately prove useful for stratifying disease stage, estimating injury severity, or identifying patients more likely to benefit from targeted neurorestorative interventions. What remains necessary is stronger analytical standardization, more rigorous target validation, and delivery platforms capable of overcoming central nervous system barriers with acceptable specificity and safety (Paul and Sullivan 2019) (Table 3).

**TABLE 3:** Translational perspectives on miRNA biomarkers and therapeutic strategies.

Translational goal	Strategy	Why it is attractive	Main barriers	Practical notes/examples mentioned	Reference
Biomarkers for disease stage or severity	Measure circulating miRNAs in blood, including protein‐bound and extracellular vesicle‐associated forms	miRNAs can remain relatively stable in blood and may reflect tissue stress or disease activity	Disease heterogeneity, inter‐laboratory variation, treatment effects, sampling time, RNA isolation differences, platform‐dependent quantification	Requires harmonized pre‐analytical and analytical workflows before differential expression can be interpreted as a robust disease signal	(Zhao et al. 2016; Huber et al. 2023)
Therapeutic inhibition when a miRNA is pathologically elevated	Anti‐miR or antagonist‐based suppression	Can directly reduce an overactive regulatory signal linked to disease pathways	One miRNA can influence many mRNAs, creating off‐target risk; brain delivery remains particularly difficult	Conceptual example: suppressing an overexpressed miRNA in Alzheimer's disease or another CNS disorder	(Hochrein et al. 2018; Khuu et al. 2016)
Therapeutic replacement when a beneficial miRNA is reduced	miRNA mimic or synthetic oligonucleotide replacement	May restore regulatory balance in pathways disrupted by aging or disease	Risk of unintended repression across multiple transcripts, uncertain tissue specificity, dose optimization challenges	Conceptual approach: replacing a depleted miRNA to normalize downstream control	(Nogimori et al. 2019; Kraus et al. 2025)
Indirect modulation through sponges or decoys	Use miRNA response elements or competing sequences to sequester miRNAs	May preserve translation of functionally important mRNAs without directly altering miRNA transcription	Sponge effects can extend beyond the intended target network, making net biological outcomes difficult to predict	Example: Rpph1 competes for miR‐326‐3p and miR‐330‐5p, increasing CDC42 translation and dendritic spine formation	(Cai et al. 2017; Jafarzadeh et al. 2022)
Study design in neurological disease	Interpret miRNA shifts alongside treatment status and disease stage	Improves biological interpretation and reduces confounding	Misclassification of therapy‐induced changes as disease‐specific signals	Especially important in acute brain injury, where sampling before versus after intervention can produce very different miRNA profiles	(Wu and Chen 2016; Kong et al. 2025)
Translation toward CNS‐directed therapy	Develop targeted delivery systems such as vectors, nanoparticles, or cell‐specific platforms	High relevance for neurodegeneration and neurorestoration	Blood–brain barrier restriction, limited cell‐type specificity, immune activation, systemic toxicity, uncertain long‐term safety	Brain delivery remains a major bottleneck and will likely determine clinical feasibility	(Anwarkhan et al. 2024; Chauhan and Jain 2025)

## 14 Future Directions

Mapping miRNA‐target networks has substantially expanded our understanding of aging mechanisms across cellular, tissue, and organismal levels. At the same time, efforts to define age‐associated miRNAs have helped connect core aging biology with the emergence of age‐related disease (Turko et al. 2025). A major lesson from this work is that aging hallmarks do not operate independently. Telomere attrition, for example, is closely linked to cellular senescence, while senescence intersects with DNA damage signaling, inflammatory remodeling, and the senescence‐associated secretory phenotype. This interdependence means that miRNA‐mediated perturbation of one hallmark may propagate across several others, which is particularly relevant in the aging brain, where plasticity, inflammatory tone, metabolic resilience, and repair capacity are tightly coupled (Ma et al. 2025; Farr and Almeida 2018).

Among the miRNAs repeatedly implicated in aging, miR‐34a stands out because it has been associated with age‐related changes in both human and experimental datasets. This recurring pattern has led to interest in miR‐34a as a candidate biomarker of aging‐related dysfunction in the brain and, in some studies, the heart (Moravčík et al. 2023). In cardiac aging, miR‐34a has been linked to telomere attrition, DNA damage signaling, cardiomyocyte apoptosis, and impaired recovery after myocardial infarction. In the brain and in Alzheimer's disease‐related settings, miR‐34a has been discussed in connection with genes involved in activity‐dependent plasticity, myelin‐associated adaptation, angiogenic support linked to neural activity, and pathways relevant to memory decline and neurodegeneration (Zhao et al. 2018). For a brain‐focused aging framework, the most important unresolved question is not simply whether miR‐34a changes with age, but which neural cell types, brain regions, and pathological contexts are most sensitive to its regulatory effects. This tissue‐ and cell‐specific uncertainty remains a major mechanistic gap.

One plausible explanation for age‐dependent induction of miRNAs such as miR‐34a involves promoter‐level regulation by stress‐responsive transcription factors. The miR‐34a promoter has been reported to contain binding sites for p53, NF‐κB, and STAT3, all of which can become activated in aging tissues under conditions of oxidative stress and chronic low‐grade inflammation (Raucci et al. 2021; Shi et al. 2020). This is mechanistically important because it places miR‐34a within broader regulatory circuits rather than treating it as an isolated marker. In particular, the p53/miR‐34a/SIRT1 axis provides a plausible framework linking DNA damage, senescence‐associated signaling, and metabolic adaptation. If this model is confirmed more rigorously in brain aging, it could help explain why a limited number of miRNAs repeatedly emerge across multiple hallmarks.

These observations also strengthen the rationale for therapeutic strategies aimed at modulating pathogenic miRNA activity. Antisense oligonucleotides remain the leading platform for miRNA inhibition, and several chemical modifications, including 2′‐O‐methyl, 2′‐MOE, and locked nucleic acid chemistries, have been used to improve stability, nuclease resistance, and binding affinity (Li et al. 2025; Shen et al. 2019). The challenge is not only to enhance potency but also to preserve acceptable safety, since improved affinity can be accompanied by altered toxicity, immune activation, or off‐target effects. This balance is especially important in the central nervous system, where long‐term exposure and limited tissue accessibility complicate translational design.

Most therapeutic development in the miRNA field has so far focused on peripheral disease because delivery is more feasible outside the brain. Examples explored in preclinical or early translational settings include inhibition of miR‐122 in hepatitis C‐related programs, miR‐21 in renal fibrosis, miR‐33 in atherosclerosis, miR‐92 in peripheral artery disease, and miR‐15 in myocardial infarction. Early clinical development has also included miRNA mimic strategies, including a miR‐34a mimic evaluated in liver cancer‐related settings, as well as antisense approaches directed against miR‐122 (Hua et al. 2021; Farooqi et al. 2016). These studies support the feasibility of miRNA‐directed intervention as a therapeutic class, but they also highlight how far the field still is from reliable CNS application. Blood–brain barrier penetration, neural cell‐type targeting, sustained efficacy, and safety remain the major barriers to clinical translation in neurodegenerative disease and neurorestoration.

Future work would benefit from three priorities: first, stronger evidence grading that clearly separates prediction, association, and direct functional validation; second, greater emphasis on brain‐region and cell‐type specificity; and third, translational platforms capable of delivering miRNA modulators across the blood–brain barrier with high precision. Progress in these areas will determine whether miRNAs remain primarily informative biomarkers of brain aging or become actionable therapeutic targets.

## 15 Conclusion

Aging is not a single linear process but a multilayered biological transition that progressively reshapes genomic stability, nutrient sensing, proteostasis, mitochondrial function, stem cell behavior, telomere maintenance, epigenetic regulation, and intercellular communication. Across these domains, miRNAs emerge as compact but influential regulatory nodes capable of converting relatively modest expression changes into broader functional consequences. Because each miRNA can regulate multiple transcripts simultaneously, age‐associated shifts in miRNA abundance may reprogram interacting signaling networks rather than isolated pathways.

In the aging brain, transcriptomic and regulatory evidence points to a coordinated decline in genes involved in plasticity, repair, metabolic support, and adaptive maintenance, together with a rise in immune and inflammatory programs often discussed under the concept of inflammaging. miRNAs are well positioned within this transition because they can influence DNA repair pathways, mitochondrial homeostasis, synaptic plasticity, myelin‐related adaptation, endothelial integrity, and inflammatory signaling at the same time. Recurrently discussed candidates such as miR‐34a and miR‐146a illustrate this integrative role, although the level of evidence is not uniform across all proposed targets and should be interpreted according to whether support comes from prediction, expression association, or direct functional validation. miRNA regulation also offers a plausible molecular link between cell‐intrinsic aging and systemic influences. Circulating factors, extracellular vesicles, and exosome‐associated miRNAs may contribute to age‐related signaling between tissues and may help explain why young and aged systemic environments exert opposing effects on plasticity, cognition, vascular stability, and recovery potential. This systems‐level dimension is especially relevant to brain aging, where blood–brain barrier function, endothelial responses, glial activation, and peripheral immune cues interact continuously rather than independently.

From a translational perspective, miRNAs remain attractive as both biomarkers and therapeutic candidates. Their relative stability in biofluids supports their value for minimally invasive monitoring, yet disease heterogeneity, methodological variability, treatment‐related confounding, and incomplete target validation remain substantial barriers to clinical implementation. Therapeutic strategies such as anti‐miRs, mimics, and miRNA sponges have demonstrated conceptual promise, but network complexity, off‐target effects, dose control, and efficient delivery to the central nervous system continue to limit progress. What now seems most important is not simply cataloging additional age‐associated miRNAs, but determining which miRNAs are truly functional drivers in brain aging, in which cell types they act, and under what conditions they become therapeutically tractable. A more rigorous brain‐centered framework that combines mechanistic validation, evidence grading, and cell‐specific translational design will be essential if miRNA biology is to move from descriptive association toward meaningful application in neurodegeneration and neural recovery.

## Author Contributions

**Manoj Kumar Mishra**: writing – review and editing. **Vandana Tripathi**: writing – review and editing. **Omayma Salim Waleed**: writing – review and editing. **Rajashree Panigrahi**: writing – review and editing.

## Funding

The authors have nothing to report.

## Conflicts of Interest

The authors have no relevant financial or non‐financial interests to disclose.

## Data Availability

Data sharing is not applicable to this article as no datasets were generated or analyzed during the current study.
